# Smartphone-based Anemia Screening *via* Conjunctival Imaging with 3D-Printed Spacer: A Cost-effective Geospatial Health Solution

**DOI:** 10.2174/0115734056389602250826081355

**Published:** 2025-08-29

**Authors:** A.M. Arunnagiri, M. Sasikala, N. Ramadass, G. Ramya

**Affiliations:** 1 Department of Biomedical Engineering, College of Engineering Guindy, Anna University, Chennai, 600025, Tamil Nadu, India; 2 Department of Electronics and Communication Engineering, College of Engineering Guindy, Anna University, Chennai, 600025, Tamil Nadu, India

**Keywords:** Anemia,3D printed spacer, Eye conjunctiva images, YOLOv8, SAM, Multi-layer perceptron, Android mobile application

## Abstract

**Introduction:**

Anemia is a common blood disorder caused by a low red blood cell count, reducing blood hemoglobin. It affects children, adolescents, and adults of all genders. Anemia diagnosis typically involves invasive procedures like peripheral blood smears and complete blood count (CBC) analysis. This study aims to develop a cost-effective, non-invasive tool for anemia detection using eye conjunctiva images.

**Method:**

Eye conjunctiva images were captured from 54 subjects using three imaging modalities such as a DSLR camera, a smartphone camera, and a smartphone camera fitted with a 3D-printed spacer macro lens. Image processing techniques, including You Only Look Once (YOLOv8) and the Segment Anything Model (SAM), and K-means clustering were used to analyze the image. By using an MLP classifier, the images were classified as anemic, moderately anemic, and normal. The trained model was embedded into an Android application with geotagging capabilities to map the prevalence of anemia in different regions.

**Results:**

Features extracted using SAM segmentation showed higher statistical significance (p < 0.05) compared to K-Means. Comparing high resolution (DSLR modality) and the proposed 3D-printed spacer macrolens shows statistically significant differences (p < 0.05). The classification accuracy was 98.3% for images from a 3D spacer-equipped smartphone camera, on par with the 98.8% accuracy obtained from DSLR camera-based images.

**Conclusion:**

The mobile application, developed using images captured with a 3D spacer-equipped modality, provides portable, cost-effective, and user-friendly non-invasive anemia screening. By identifying anemic clusters, it assists healthcare workers in targeted interventions and supports global health initiatives like Sustainable Development Goal **(**SDG) 3.

## 1 INTRODUCTION

Anemia is a medical condition characterized by a decrease in the number of red blood cells or the hemoglobin concentration in the blood. This results in a reduced capacity to transport oxygen to body tissues, often leading to symptoms such as fatigue, weakness, dizziness, and shortness of breath. Vulnerable populations, including pregnant women and children, are at increased risk, and in severe cases, anemia can be fatal [1]. According to WHO data from 2021, anemia affects 31.2% of women and 17.5% of men globally, with women of reproductive age disproportionately impacted [2]. Conventional diagnosis relies on complete blood count (CBC) testing, an invasive and often expensive procedure that requires clinical infrastructure and trained personnel. Such limitations hinder frequent monitoring, especially in remote and resource-constrained settings. Clinically, a hemoglobin level below 10 g/dL is typically considered anemic, while normal ranges are 12–15.5 g/dL for women and 13.5–17.5 g/dL for men [3]. Among physical signs, pallor of the conjunctiva, nail beds, and palms is widely used for anemia assessment. Studies have shown that conjunctival pallor is among the most consistent indicators, independent of racial skin tone [4-11]. This makes the conjunctiva an attractive site for non-invasive image-based anemia screening.

Recent years have seen growing interest in non-invasive anemia detection, with technologies ranging from smartphone cameras to advanced image analysis tools [12, 13]. Early approaches used digital photography and manual assessment of conjunctival pallor, followed by developments using optical sensors and photoplethysmography to estimate hemoglobin levels without the need for blood samples [14-16].

Mobile-based methods began gaining traction in the 2010s. For instance, apps were developed to analyze conjunctival redness and estimate hemoglobin levels under various lighting conditions using regression models [17, 18]. Simultaneously, deep learning models began to be employed for real-time object detection and segmentation. The YOLO algorithm, originally used in industrial applications like helmet detection [19], demonstrated strong potential in medical imaging. Segmentation techniques such as K-means clustering and fuzzy C-means were applied for identifying regions of interest in medical and agricultural imaging tasks [3, 20, 21]. For conjunctival analysis, the combination of segmentation and machine learning classification enabled improved performance in detecting anemia. Edge detection, SLIC superpixels, and neural networks were also employed to extract features from eye images [22, 23]. These systems, while innovative, often relied on high-resolution DSLR cameras and manual input, limiting scalability and usability in real-world settings. Moreover, variability in image capture due to ambient light and inconsistent camera positioning further reduced accuracy. In recent years, artificial intelligence (AI) has emerged as a pivotal tool across various domains of medicine, including radiology, dermatology, ophthalmology, and pathology. AI plays a particularly transformative role in medical imaging by enabling automated detection, segmentation, and classification of clinically relevant features with high speed and accuracy. The integration of AI with mobile health (mHealth) technologies enables real-time, on-device inference, thereby facilitating accessible and early diagnosis in resource-limited settings. These advancements highlight the growing significance of AI-driven mobile solutions in enhancing public health surveillance and supporting community-level clinical interventions [24-27]. Despite these advancements, their application in anemia screening remains limited.

A major gap in the literature is the lack of a standardized, cost-effective, and fully automated system for non-invasive anemia screening using conjunctival images. Despite promising results from various algorithms, few solutions have addressed the need for portable, user-friendly, and accurate tools deployable in field settings.

This study presents a novel approach using a smartphone camera equipped with a 3D-printed spacer and macro lens to standardize image acquisition. The fixed 45 mm distance between the lens and the eye ensures consistent illumination and focus, minimizing user variability. We integrate You Only Look Once (YOLOv8) for real-time conjunctival localization and the Segment Anything Model (SAM) for high-precision segmentation. Extracted features are used in a multi-layer perceptron (MLP) classifier to predict anemia status. The entire pipeline is embedded in an Android application with geotagging capabilities, enabling real-time mapping of anemia prevalence for targeted public health interventions. This work demonstrates a scalable, cost-effective, and clinically relevant solution for anemia screening in resource-limited settings.

## 2 MATERIALS AND METHODS

Using the insights from the literature, the research suggests a six-stage method. The six stages of the algorithm, which comprise image acquisition, ROI extraction, segmentation, feature extraction, classification, and the development of mobile applications, are depicted in the block diagram in Fig. (**1**).

### 2.1 Image Acquisition

The quality of the images gathered determines how well the model performs. Acquiring images is the first and most crucial stage in the entire process. A total of 54 participants (24 males and 30 females), aged between 19 and 49 years, were enrolled in the study. Prior to data collection, all participants were thoroughly informed about the research objectives and procedures, and written informed consent was obtained from each of them. The study was conducted in accordance with the ethical principles outlined in the Declaration of Helsinki. The data collection followed a standardized protocol and was approved by the Institutional Ethics Committee of Srinivasan Medical College and Hospital (IEC No: 34/23, dated 04.10.2023). Data collection involved a set of protocols as follows: All subjects were between the ages of 19 and 49. The distance between the camera and the eye ranged approximately from 4 to 7 cm, with an angle of 40-45 degrees. The experimental protocol for data collection was carried out in such a way that informed consent was obtained from all participants. Multiple eye images were captured from both eyes of 54 subjects using three different modalities of cameras, such as a DSLR (Canon EOS 200DII), a Mobile phone (one plus 8T), and a 3D printed spacer Macro lens fitted with the Mobile phone camera(one plus 8T). The Hemoglobin (Hb) level of each subject is measured using CBC analysis. Thus, Hb value measured is used as a gold standard for diagnosing and labeling the eye conjunctiva images acquired from the corresponding subject.

Based on Hb value, eye images are labelled as normal, moderately anemic, and anemic concerning the range of their Hb values, respectively, and it is represented in Table **1** following WHO guidelines [3]. The protocol for image acquisition consists of two criteria: inclusion criteria and exclusion criteria. In the inclusion criteria, all participants aged between 19 to 49 years with minimal stress, a sound sleep pattern, and clear eyes are considered. Sleep deprivation, conjunctivitis, and alcohol addiction are some of the exclusion criteria for the research work. Three different modalities have been used for image acquisition, namely Digital Single-Lens Reflex, also known as DSLR Camera (Canon-II EOS type), mobile phone camera (OnePlus 8T), and 3D Printed Spacer Macrolens fitted with Mobile phone camera (OnePlus 8T). This approach has been adopted to investigate the image resolution difference among the modalities for capturing the eye conjunctiva images. Laboratory hemoglobin measurement taken from participants by complete blood count (CBC) analysis within four hours of capturing eye conjunctiva images. Based on Hb value, eye images are labelled as anemic, moderately anemic, and normal concerning the range of their Hb values, respectively. The intention of the present study is to screen anemia using an acquisition device or a modality that is low-cost and gives accurate predictions on par with an acquisition device having superior resolution, such as a DSLR Camera. Hence, an in-house 3D printed spacer macrolens fitted with a mobile phone camera is used as a low-cost modality to capture eye conjunctiva images, and its predictions are investigated. Before image acquisition with each modality, a DGK Digital Kolor Pro 16:9 ColorChecker was used for color calibration to ensure consistency across devices.

#### 2.1.1 Devices used for Image Acquisition

DSLR camera (Canon-II EOS type) has a 24.1-megapixel APS-C CMOS sensor that can capture images at a resolution of 6000 x 4000 pixels. The OnePlus 8T smartphone camera, which has an IMX586 sensor, is the second camera modality used in the study. It can capture digital images in RAW mode at a resolution of 3000 x 4000 pixels, ensuring that the resolution of the image is unaffected by the phone’s processor. A 3D-printed Spacer Macro lens equipped with a smartphone assembly serves as the third mode. The assembly comprises a mobile phone, a 20x macro lens (specifically the SKYVIK SIGNI X macro lens), and an indigenously designed 3D-printed spacer. Fig. (**2a**) shows the Front view of the 3D printed Spacer fabricated using Polyethylene terephthalate glycol (PETG) material using a Dreamer 3D printer. Polyethylene terephthalate is a thermoplastic material resistant to heat, impact, and solvents. It is used in advertising displays and electronic insulators. It is easily recyclable and prevents pollution to the environment. 3D printed PETG-based spacer lens is designed in such a way that the Macro lens can be fitted at the smaller diameter end of the spacer, where the dimensions are shown in Fig. (**2b**). Thus, making it portable for all mobile phone cameras to be attached. The macro lens is attached to a mobile camera (OnePlus 8T) and assembled into the 3D-printed spacer, which is positioned 45 mm away from the subject’s eye as shown in Fig. (**2b**) Overall 3D printed spacer from the top field of view is shown in Fig. (**2c**) The developed 3D printed spacer-based optical assembly system is registered under the Indian Patent Act with application number 202441088606. Image acquisition using DSLR camera modality is shown in Fig. (**3a-c**) of anemic, moderate anemic, and normal subjects, respectively. Anemic, moderately anemic, and anemic eye conjunctiva images are depicted in Fig. (**3d-f**) by photos taken using a mobile phone camera. Fig. (**3g-i**) illustrates the eye images captured using a 3D-printed spacer Macro lens attached to a mobile phone camera for anemic, moderate anemic, and normal subjects, respectively. One of the advantages of using 3D printed spacer modality is the constant distance between the lens and the subject’s eye, as there are more chances distance gets varied while acquiring without a spacer. A total of 611 eye conjunctiva images were taken using each of the three modalities. Of the total 611 images, 132 images are anemic class, 169 images are moderately anemic class, and 310 images are normal class. The images that were captured were classified as follows: anemic (hemoglobin level less than 10.5 g/dL for both male and female), moderate anemic (hemoglobin level 10.5 g/dL-13.5 g/dL for male and 10.5 g/dL-12 g/dL for female). For hemoglobin level 13.5 g/dL or above, and hemoglobin level 12.5 g/dL or above is labelled as Normal class for males and females, respectively as mentioned in Table **1**. Every image that was taken with various modalities is stored in the .PNG file format.

### 2.2 ROI Extraction

In image processing applications, obtaining the Region of Interest (ROI) in an image is an essential step towards achieving improved feature extraction. Therefore, the analysis may focus on the most important areas of the picture by isolating the ROI and focusing on Relevant Data, which will increase the accuracy of detection, classification, and other processing tasks.

The YOLO V8 (You Only Look Once) based Realtime object detection system is a novel approach used in this work. Employing a bounding box, it can identify the conjunctiva region of the eye.

#### 2.2.1 YOLOv8 Object Detection

Popular object detection model YOLO (You Only Look Once) is renowned for its accuracy and quickness. Joseph Redmon *et al*. made the initial introduction of it. Object detection models may be divided into two categories: single-stage and two-stage models. Three parts make up a single-stage object detector architecture (such as YOLO): a head to produce dense predictions, a neck, and a backbone. The most recent version of You Only Look Once, known as YOLOv8, impressively prioritizes accuracy and speed when it comes to object identification. A pre-trained model of the YOLOV8-m version using the Common Object in Context (COCO) dataset with a 640 x 640 pixels input size was used to train the YOLOV8-m model in this research work. As the cornerstone, the backbone functions as an image feature extractor for the input. A customized CNN (Convolutional Neural Network) known as CSPDarknet53 is used by YOLOv8 to train. Cross-Stage Partial (CSP) connections are a unique feature incorporated into this design. The network’s accuracy and gradient flow during training are eventually improved by these connections, which also improve information flow across different network stages. In the process of combining feature maps, the Neck serves as the feature extractor. It uses data that the backbone has retrieved from various phases. This makes it possible for the network to collect data from the picture at different sizes, which is essential for precise object recognition. In contrast to YOLOv5, which uses the Feature Pyramid Network (FPN), YOLOv8 adds the C2f module, a new module. In the last stage, the head is in charge of generating predictions from the information that has been processed. It predicts bounding boxes for possible items along with their class probabilities using the combined feature maps from the neck. Tables **2**, **3** provides detailed information on the YOLO V8 parameters. Convolutional neural networks (CNNs) extract low, medium, and high-level feature maps from an input picture, forming the core of the system. Feature Pyramid Network (FPN) and other path aggregation blocks are used by the neck to integrate these feature maps. Lastly, stage activities are carried out by the model head. When feature maps are applied using anchor boxes, the final result is shown, containing classes, object scores, and bounding boxes. Additionally, YOLOv8 employs a cutting-edge method known as Anchors Plus, which modifies the anchor boxes following the image’s input form. Better performance is achieved with varying input sizes as a consequence. The utilization of a hybrid backbone architecture in YOLOv8 provides an additional enhancement by merging elements from the CSP Darknet and Efficient Net models. The overall architecture block diagram is shown in Fig. (**4**).Thus, object recognition is more precise since the model can collect both high-level and low-level data. As seen in Fig. (**5**), YOLOv8 functions as a single-stage detector overall with quicker inference times and more accuracy.

### 2.3 SEGMENTATION

Both supervised and unsupervised learning are used to accomplish the segmentation approaches. Two segmentation approaches have been explored in this work, namely, Segment Anything Model (SAM) and K-means clustering. The Segment Anything model is used for supervised learning, while Color-based k-means clustering is used for unsupervised learning. The segmentation approach is shown in Fig. (**6**).

#### 2.3.1 Color-based K-means Clustering

An unsupervised machine learning method called color-based k-means clustering derives structures or patterns from incoming data without the need for labelled answers. During the clustering stage, the RGB color space of the input picture is further converted into a CIE Lab color model (introduced by the Commission Internationale de l’Eclairage) (L*a*b*) [20, 28]. The chromaticity-layer b* (which displays the color distribution along the blue-yellow axis), the chromaticity-layer a* (which displays the color distribution along the red-green axis), and the luminosity layer L* are the layers that make up the L*a*b* space [7, 9, 20]. Each of the two levels (a* and b*) has all of the necessary color information. As a result, the method for classifying the colors in the a*b* space uses K-means clustering [7, 9, 21]. Following the process of clustering, the major segment is chosen from among the clusters that comprise an area of interest. The clusters that have no connection to the area of interest are removed. The algorithm’s overall operation is shown in Fig. (**7**).

#### 2.3.2 Segment Anything Model

A network architecture called the Segment Anything Model (SAM) creates segmentation masks from pictures by using prompts. Meta AI created SAM, which was made available in April 2023. Fig. (**8**) shows the three parts of SAM: a quick mask decoder, a flexible prompt encoder, and an image encoder. SAM's architecture centers on the image encoder, which processes raw photos into a dense feature matrix using a transformer-based approach. This feature matrix forms the basis for identifying elements in the image. A key component, the prompt encoder, interprets various input cues (text, points, rough masks) and converts them into embeddings to guide segmentation. The mask decoder then combines this data to generate precise segmentation masks. The process involves the image encoder building an understanding of the image, the prompt encoder providing context, and the mask decoder producing the final segmented output based on the input prompts.

### 2.4 Feature Extraction

Complex data is simplified with the use of feature extraction. As a result, models are more accurate in predicting anemic situations when color and statistical data from Segmented Eye Conjunctiva Region data are utilized to identify the most essential facts.

#### 2.4.1 Statistical Features

##### 2.4.1.1 Mean

Mean represents average pixel intensity in an image, calculated by adding intensities and dividing by the overall number of pixels, indicating brightness and central tendency. The representation of the mean is given in eq. (**1**)

**Table:** 

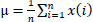	(1)

The ratio of the mean red and green channels in an image represents the average intensity or brightness relationship between the red and green color channels within an image. The equation is given by eq. (**2**).

**Table:** 

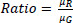	(2)

The variation in brightness or intensity between the red and green color channels within an image is represented by the difference between the mean red and green channels. The equation is given by eq. (**3**).

**Table:** 

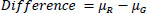	(3)

##### 2.4.1.2 Standard Deviation

Standard deviation measures the variation in pixel intensity around the mean, indicating the extent to which each value differs from the average. In contrast to a larger standard deviation, which denotes a wider range, a smaller standard deviation implies that the values are closely grouped. The mathematical equation is given by eq. (**4**).

**Table:** 

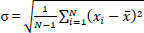	(4)

##### 2.4.1.3 Entropy

Entropy measures the uncertainty in pixel intensities in an image, providing details on the intricacy of the distribution. It is often estimated using the histogram of pixel intensities, with higher entropy values indicating a more complicated distribution and lower entropy values indicating a more uniform distribution. The mathematical equation is given in eq. (**5**).

**Table:** 

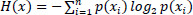	(5)

#### 2.4.2 Color-based Features

##### 2.4.2.1 High Hue Ratio

An HSI color space is created from the RGB picture. Hue, saturation, and intensity are represented by the letters H, S, and I in the HSI color model, respectively. Next, a characteristic known as the high hue ratio (HHR), or the percentage of pixels in the image’s hue component with high values, is retrieved. The formula for figuring out the High Hue Ratio (HHR) is found in eq. (**6**).

**Table:** 

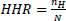	(6)

##### 2.4.2.2 Red pixel Percentage

The percentage of pixels in a picture that are classed as red of all of the pixels in the image is known as the red pixel percentage. The percentage indicates the portion of the image that is made up of red pixels. Eq. (**7**) may be used to get the percentage of red pixels in a picture by counting the number of red pixels and determining the total number of pixels in the image.

**Table:** 

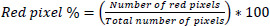	(7)

### 2.5 Classification

#### 2.5.1 Multi-layer Perceptron

Any input dimension may be converted to the required dimension using a multi-layer perceptron, which is a completely linked dense layer. A neural network with hidden layers is called a multi-layer perception. However, this network only makes use of one completely linked layer. A large number of processing components are combined to create the network, which has weighting functions that may be adjusted for each input. These processing components are often arranged in a layer-by-layer configuration with complete or haphazard connections between the levels [29]. In general, there are three or more levels: an input layer where data are sent to the network *via* an input buffer, an output layer with a buffer containing the output response to a given input, and one or more intermediate or hidden layers [18]. The implementation of the MLP in this work was carried out using Python 3.9 (Python Software Foundation, Wilmington, DE, USA). Table **4** lists the overall model parameters utilized in the study. Fig. (**9**) displays the neural network arrangement’s architecture.

#### 2.5.2 Performance Metrics

The performance of the classifier is evaluated using metrics such as accuracy, precision, sensitivity, and specificity for anemic, moderate anemic, and normal classifications; the related results are displayed from equations (**8** to **12**), where TP denotes True positive, FP denotes False positive, TN denotes True negative, and FN denotes False Negative, respectively. With diagonal components denoting accurate predictions and non-diagonal elements denoting inaccurate forecasts, it depicts the actual class and the predicted class. The performance of classification models is estimated using these metrics, the majority of which are based on the model’s prediction using dataset values.

The AUC stands for area under the ROC. The main feature of AUC lies in its ability to measure the quality of a predictor irrespective of the decision threshold. It summarizes the trade-off between the true positive rate (TPR) and false positive rate (FPR) across all possible thresholds, thus providing a comprehensive metric for model performance.

**Table:** 

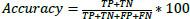	(8)

**Table:** 

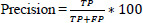	(9)

**Table:** 

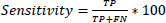	(10)

**Table:** 

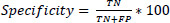	(11)

**Table:** 

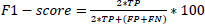	(12)

### 2.6 Mobile Application

To make it simpler for medical professionals to screen anemic patients, an Android mobile application was created using the Android Studio program [30]. This application links Google Maps with Firebase storage, which holds the information of a trained model consisting of predicted output, using which the subject’s location is identified. A flow diagram for developing Android applications may be seen in Fig. (**10**).

## 3 RESULTS AND DISCUSSION

The YOLOv8 algorithm effectively detects regions of interest by generating bounding boxes and confidence scores, reliably localizing the conjunctiva region for segmentation, as shown in Fig. (**11a**-**c**). Training performance and model metrics, including box loss and mean average precision (mAP), are depicted in Fig. (**12a-d**). The model's accuracy in predicting the position and dimensions of bounding boxes is measured using box loss, with training and validation losses recorded at 0.96 and 0.67, respectively. A lower box loss indicates better localization accuracy.

The mAP metric assesses the object detection performance of YOLOv8. It is computed as the ratio of true positives to the sum of true and false positives. The (mAP50) results are shown in Fig. (**12c**), while mAP(50–95), representing an average over multiple Intersection over Union (IoU) thresholds, is shown in Fig. (**12d**). From these figures, it is evident that the validation loss rapidly converges toward zero, suggesting that the model effectively learns meaningful and generalizable features for accurate conjunctiva localization.

Subsequently, the segmented conjunctiva regions were processed using two methods: K-means clustering (k = 3) and the Segment Anything Model (SAM). While K-means clustering was used alongside YOLOv8 to segment regions based on color similarity Fig. (**13a**-**c**), SAM provided supervised segmentation masks Fig. (**13d**-**f**). Images were acquired using three imaging modalities: DSLR camera, smartphone camera, and smartphone camera equipped with a 3D-printed spacer macro lens. To simulate real-world environments, segmentation was tested under six lighting conditions-tungsten, cloudy, daylight, flash, fluorescent, and shade-as depicted in Fig. (**14a-f**). This data augmentation increased the dataset size from 611 to 3,666 images, distributed as 792 anemic, 1,014 moderately anemic, and 1,860 normal-class images. The extracted features reflected physiological changes associated with anemia. Red Pixel Percentage and High Hue Ratio (HHR) quantified conjunctival redness, which diminishes in anemic subjects due to reduced hemoglobin and capillary perfusion. Mean Intensity and Standard Deviation captured brightness and homogeneity, while Entropy reflected textural complexity loss in anemic conjunctiva. Feature analysis revealed that HHR and red pixel percentage were highest in normal images and decreased progressively in moderate and anemic classes. Features extracted from segmented images using two segmentation approaches-SAM and K-Means-show higher statistical significance (p < 0.05) for SAM segmentation. In this study, three different imaging modalities were used. To ensure fair comparison across modalities, statistical analysis was conducted using features extracted *via* SAM. The results demonstrated statistically significant differences (p < 0.05) between DSLR and 3D spacer-based smartphone images, confirming SAM’s effectiveness across device types. The classification model was trained using 90% of the images, with 10% reserved for testing *via* a resubstitution validation strategy.

The confusion matrices corresponding to three imaging modalities-DSLR camera, mobile phone camera, and mobile phone camera fitted with a 3D-printed spacer macro lens-are presented in Fig. (**15a**-**c**) using the K-means segmentation method. Similarly, Fig. (**16a**-**c**) illustrate the confusion matrices for the same modalities processed using the Segment Anything Model (SAM). The Positive Predictive Value (PPV), representing the proportion of correctly predicted observations within each class, is annotated in the respective figures. Likewise, the False Discovery Rate (FDR), indicating the proportion of misclassified predictions per class, is also shown in the same set of figures.

The Positive Predictive Value (PPV) obtained using the K-means segmentation approach varies across different imaging modalities. For DSLR camera-based images, the PPV values are 100% for the anemic class, 98.4% for the moderately anemic class, and 98.1% for the normal class. Images captured using a mobile phone camera fitted with a 3D-printed spacer macro lens yield PPVs of 94.1%, 97.6%, and 97.5% for anemic, moderately anemic, and normal classes, respectively. In contrast, images captured using a standard mobile phone camera result in slightly lower PPVs: 90% (anemic), 96.7% (moderate), and 94.7% (normal).

Comparatively, SAM-based segmentation demonstrates enhanced performance across modalities. For DSLR-based images, the PPV values are 95.3% (anemic), 99.2% (moderate), and 100% (normal). For images acquired using the 3D-printed spacer macro lens, PPVs are 93.5% (anemic), 97.1% (moderate), and 93.6% (normal). Mobile phone images segmented *via* SAM achieved 94.9%, 99.1%, and 100% for anemic, moderate, and normal classes, respectively. As observed in Figs. (**15**, **16**), the DSLR and 3D spacer-equipped mobile phone modalities consistently outperform the standalone smartphone camera in classifying conjunctival images into normal, moderately anemic, and anemic categories. Using Eq. (**8**), classification accuracy was calculated across modalities. For K-means segmentation, classification accuracies were 98.5% (normal), 94.9% (moderate), and 97.2% (anemic). For SAM segmentation, slightly higher accuracies were achieved: 98.8% (normal), 95.0% (moderate), and 98.3% (anemic). Additional performance metrics-including precision, sensitivity, specificity, and F1-score-were computed using Equations (**8**–**12**). ROC and AUC analyses, shown in Fig. (**17a**-**d**), further validate model performance across four experimental setups, combining two segmentation algorithms (K-means and SAM) with two imaging modalities (DSLR and 3D-spacer mobile). All configurations yielded AUC values above 0.99, underscoring the high reliability of the classification models.

From the findings of Table **5**, the SAM-based segmentation method gives an edge over the conventionally used k-means clustering method in terms of specificity, and the results are statistically significant (p<0.05) . The proposed work results are benchmarked against existing literature, as shown in Table **6**. Unlike most studies that limit classification to two categories, there is an introduction of a 'moderate anemic' category, enhancing the accuracy for individuals near the anemia threshold.

The Segment Anything Model (SAM) consistently outperformed the K-means clustering algorithm across all imaging modalities. Among the tested configurations, the DSLR-SAM model achieved the highest overall performance, with Area Under the Curve (AUC) values of 0.9994 for the anemic class, 0.9982 for the moderately anemic class, and 0.9995 for the normal class. Remarkably, the SAM model applied to mobile phone images attained an AUC of 1.0 for the normal class and above 0.993 for the anemic and moderate classes, indicating its robustness and effectiveness even when used with lower-cost imaging systems. While DSLR images showed slightly higher performance, the margin was minimal, suggesting that mobile phones equipped with 3D-printed macro lens spacers, in combination with advanced segmentation algorithms like SAM, can offer a cost-effective and reliable alternative for field-based anemia screening.

An Android-based mobile application named Anemixpert was developed using Android Studio to integrate the trained anemia prediction model. The application is designed to be compatible with all versions of the Android operating system, leveraging the global ubiquity of Android smartphones to ensure wide accessibility and ease of deployment. Users capture images of their conjunctiva using a mobile phone camera fitted with a 3D-printed spacer macro lens, ensuring standardized image acquisition. These images are then processed directly within the application using the embedded deep learning model to predict anemia status. To enhance geospatial health monitoring, the app incorporates Google Maps functionality, which utilizes user-provided data to geotag the subject's location. This information is securely stored in Firebase Cloud Storage (with a 50 GB capacity), accessible only *via* password-protected authentication. Geolocation data is shared with healthcare professionals to facilitate targeted screening of geographic clusters with high anemia prevalence. This approach enables non-invasive, portable, and real-time preclinical screening of anemia, particularly in resource-constrained and remote areas. The complete workflow of the application-based screening process is illustrated in Fig. (**18**).

Table **7** contrasts our approach with leading commercial, non-invasive anemia-screening devices. Central to our method is a low-cost, 3D-printed spacer that standardizes image acquisition by fixing the smartphone-to-eye distance at ~45 mm, thereby minimizing focus and illumination variability. Produced on any Fused deposition modelling(FDM) printer with PETG material with the minimum cost of (US $1.50), the lightweight, reusable spacer adapts to most smartphone–macro-lens combinations and enables minimally trained community health workers to capture diagnostically reliable images. This practicality-along with a candid discussion of feasibility and remaining limitations-demonstrates the spacer’s potential for scalable deployment in resource-constrained clinical settings.

## 5 LIMITATIONS AND FUTURE WORK

The study is limited by a small sample size (54 subjects) and a lack of external validation, which may affect the generalizability of results. Future work should include data from diverse geographic regions, independent datasets, and broader device testing. The mobile application, currently Android-specific, requires expansion to iOS and a comprehensive usability evaluation. Additionally, the trained model should be tested across various smartphone models to assess consistency in performance under different hardware and imaging conditions. Addressing these gaps will be essential to develop a scalable, reliable, and widely deployable diagnostic solution.

## CONCLUSION

The work proposes an efficient low-cost, portable solution for preclinical anemia screening using a 3D-printed spacer macro lens integrated with a mobile phone camera. YOLOv8 accurately detects the eye conjunctiva region, while K-means and SAM segmentation techniques enable precise feature extraction. The results, comparable to those of DSLR-based imaging, highlight the potential of mobile health tools in resource-limited settings. A handheld application further supports real-time diagnosis and geo-tagging for public health monitoring. This approach aligns with SDG 3, promoting accessible and early detection of anemia.

## Figures and Tables

**Fig. (1):**
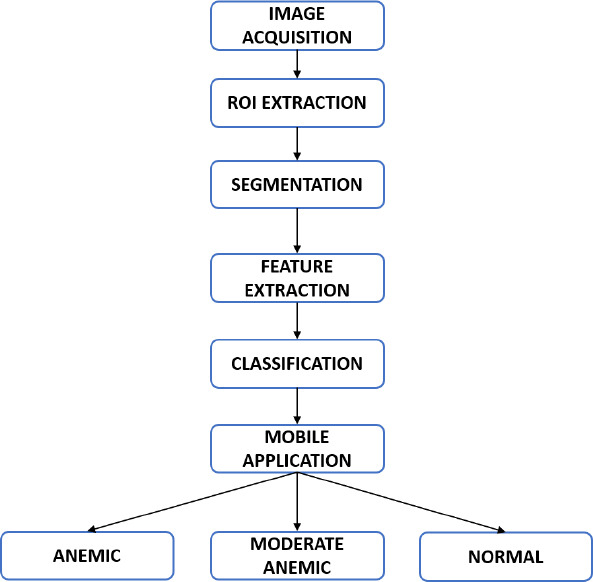
Overall Block diagram of a non-invasive approach-based anemia screening using eye conjunctiva images.

**Fig. (2a):**
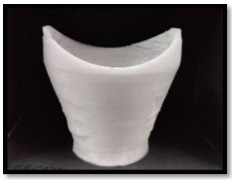
Front view of 3Dprinted spacer designed.

**Fig. (2b):**
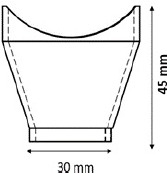
Measurements of 3D printed spacer.

**Fig. (2c):**
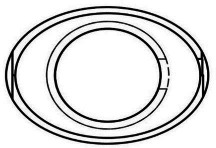
Illustration of a 3D-printed spacer in the top view.

**Fig. (3):**
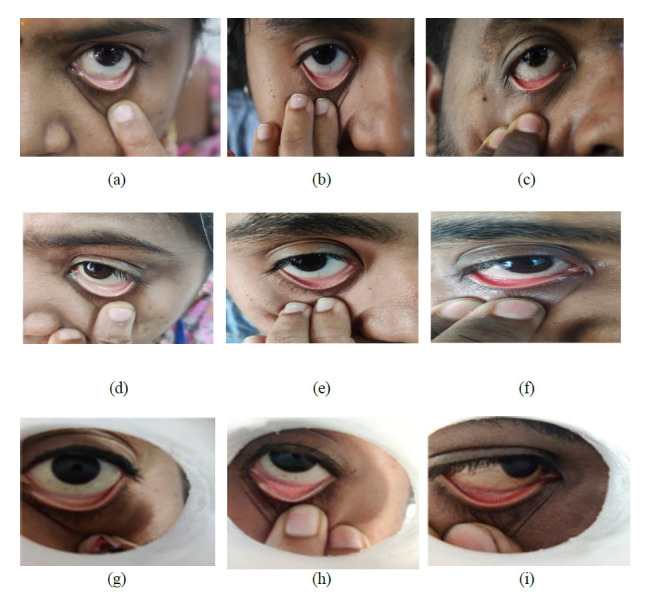
(**a**-**c**) DSLR image of Anemic subject, moderate anemic, and normal subject (left to right).. (**d**-**f**) Mobile phone camera image of Anemic subject, moderate anemic, and normal subject (left to right). (**g**-**i**) 3D printed spacer fitted Mobile phone camera image of Anemic subject, moderate anemic and normal subject (left to right).

**Fig. (4):**
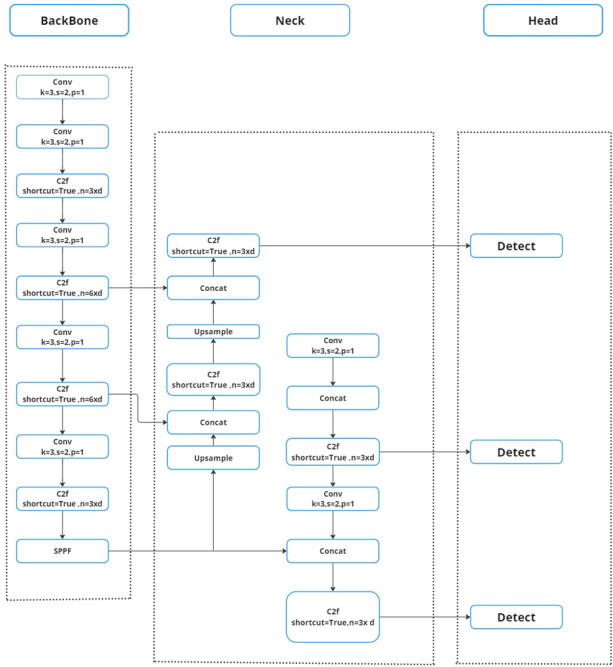
Overall block diagram of YoloV8 architecture.

**Fig. (5):**
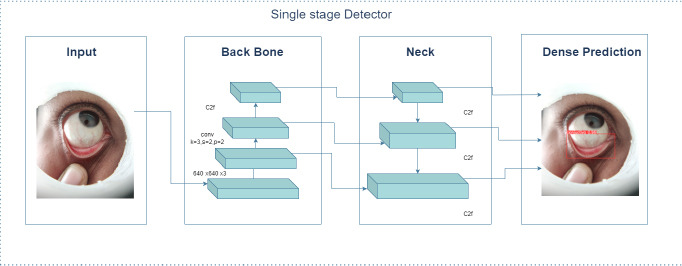
Single-stage conjunctiva region detection from eye image.

**Fig. (6):**
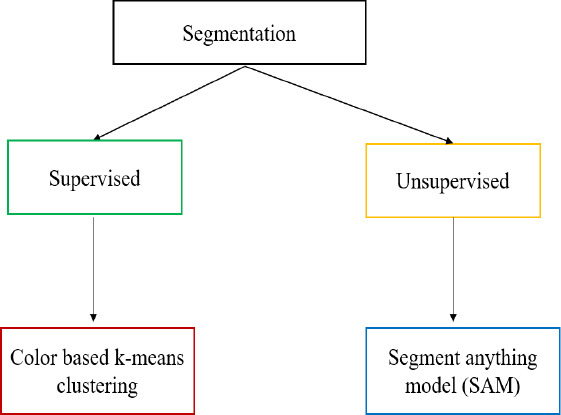
Segmentation techniques used in the extraction of conjunctiva region from the eye image.

**Fig. (7):**
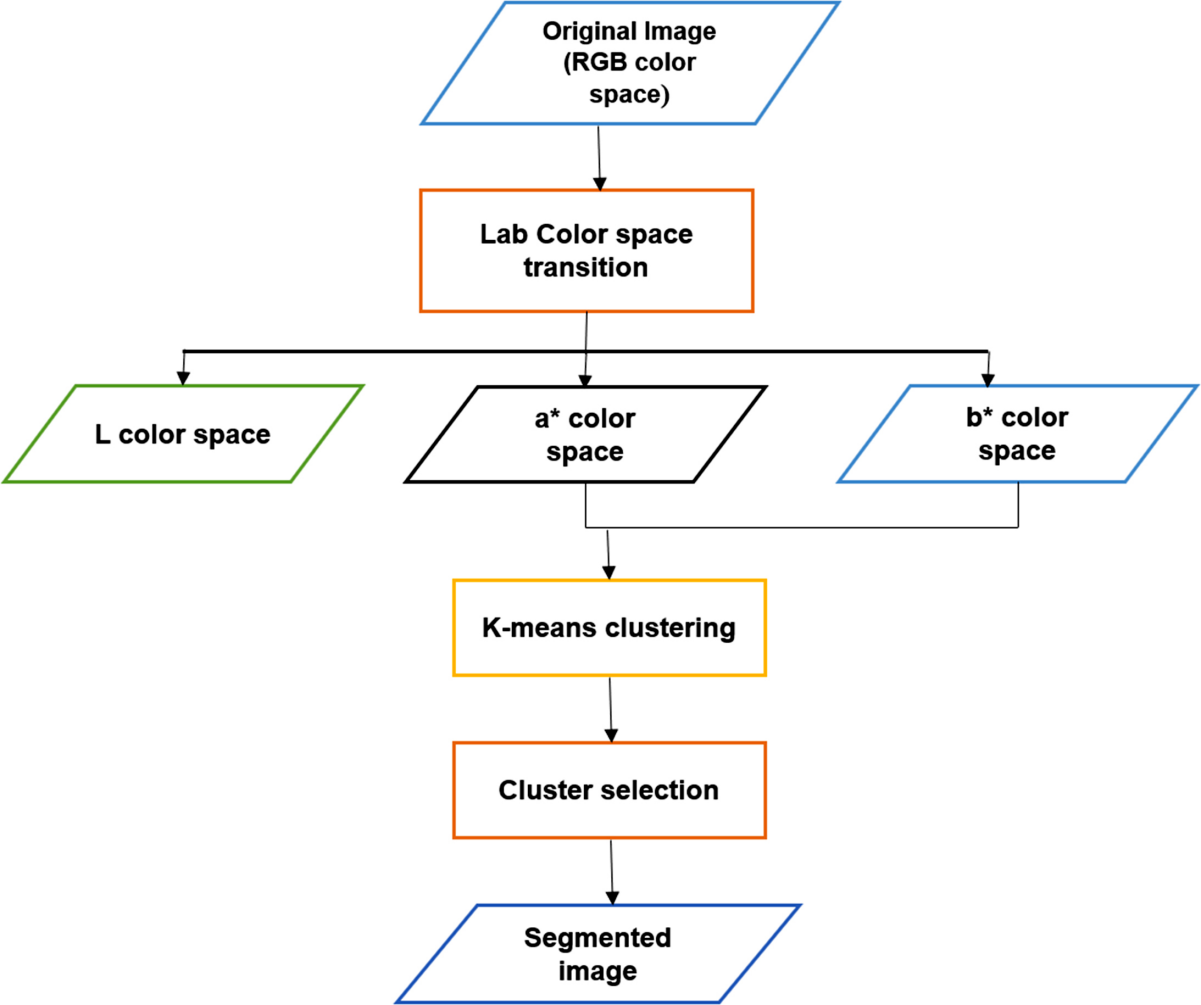
Overall flow diagram of K-means algorithm.

**Fig. (8):**
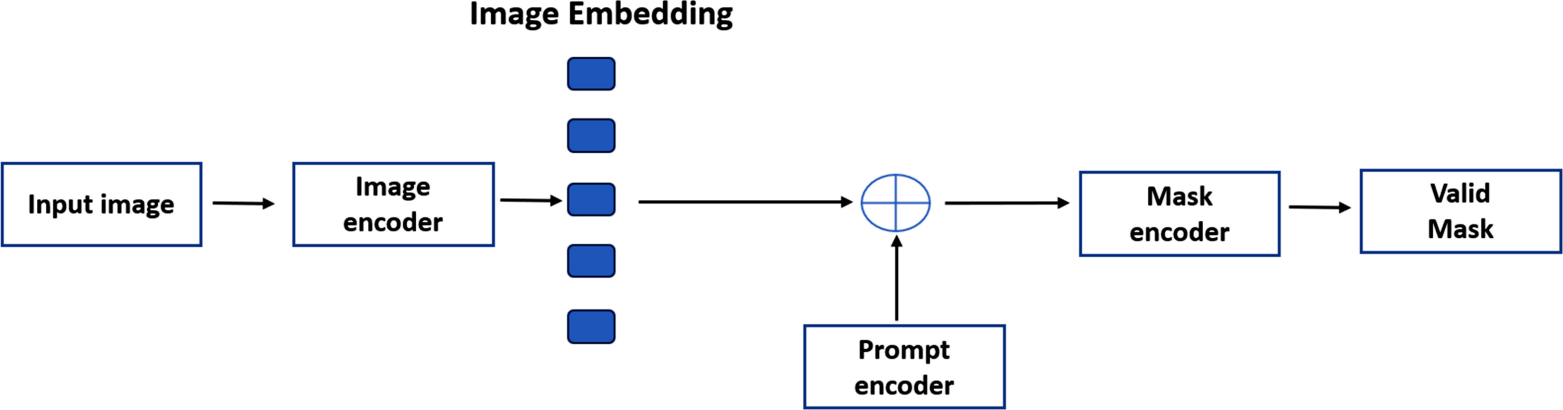
Overall flow diagram of segment anything model algorithm.

**Fig. (9):**
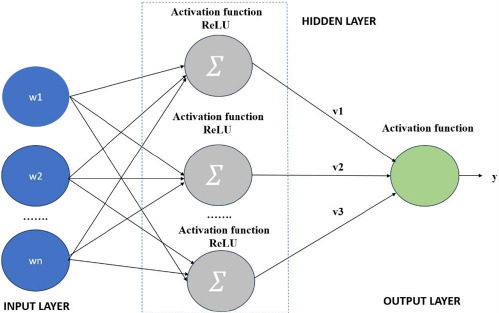
Multi-layer perceptron (MLP) architecture.

**Fig. (10):**
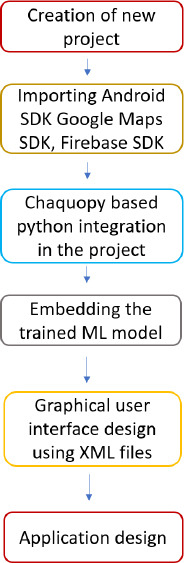
Flow diagram of android application development.

**Fig. (11a):**
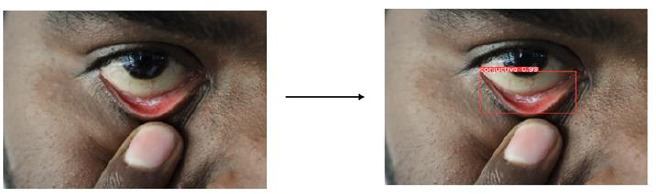
YOLO v8 Object detection for DSLR image.

**Fig. (11b):**
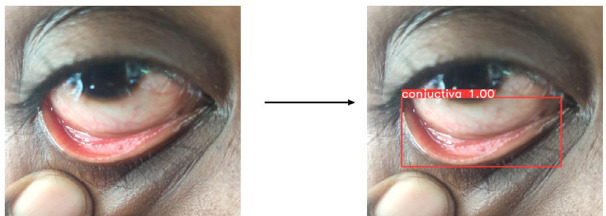
YOLO v8 Object detection for Mobile phone image.

**Fig. (11c):**
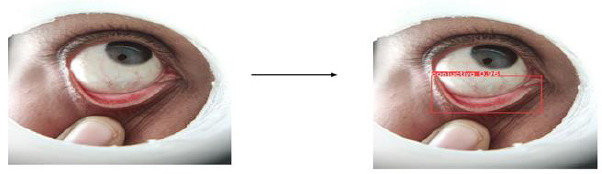
YOLO v8 Object detection for 3D-fitted Mobile phone image.

**Fig. (12):**
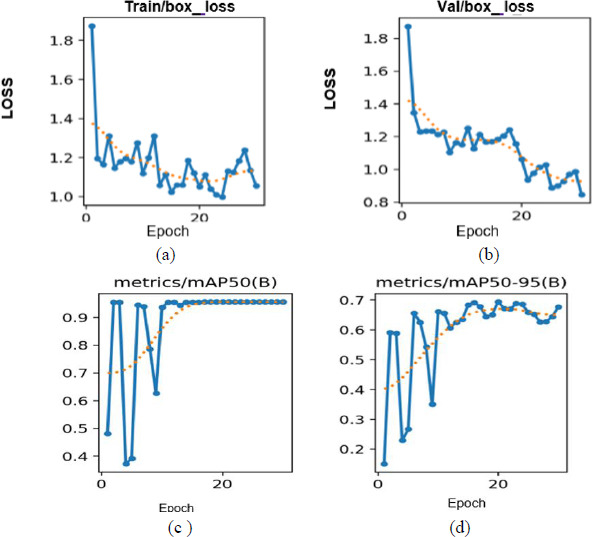
**(a)** Training graph of box loss. **(b)** validation graph of box loss. **(c)**Accuracy graph of mAP50(B). **(d)** Accuracy graph of mAP 50-95(B).

**Fig. (13a):**
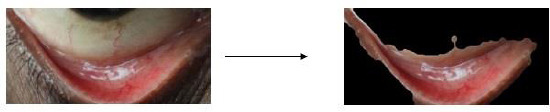
K-means clustering for DSLR image.

**Fig. (13b):**
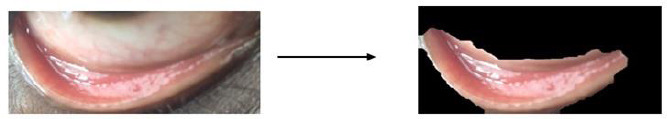
K-means clustering for Mobile phone image.

**Fig. (13c):**
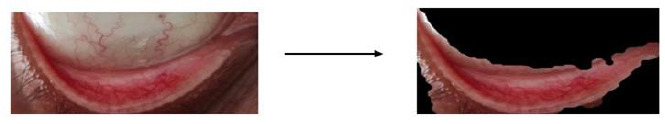
K-means clustering for 3D fitted Mobile phone image.

**Fig. (13d):**
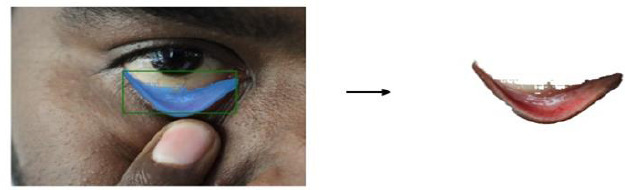
Segment Anything Model based segmentation for DSLR image.

**Fig. (13e):**
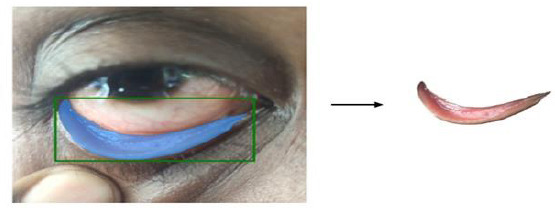
Segment Anything Model for Mobile phone image.

**Fig. (13f):**
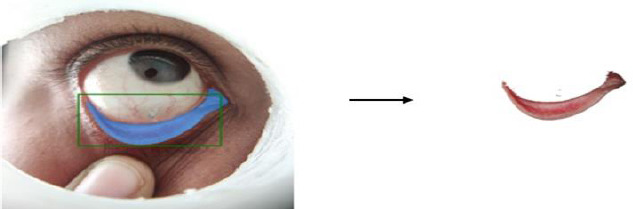
Segment Anything Model for a 3D fitted Mobile phone image.

**Fig. (14a-f):**
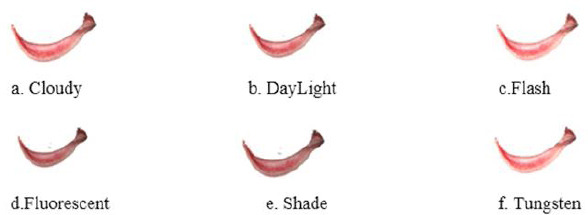
Different White balances of the extracted Eye conjunctiva region.

**Fig. (15a):**
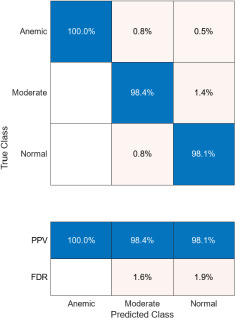
Confusion matrix of DSLR camera images using K-means.

**Fig. (15b):**
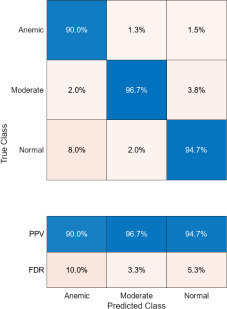
Confusion matrix of mobile phone camera images using K-means.

**Fig. (15c):**
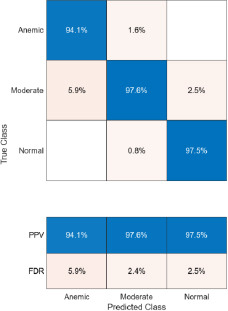
Confusion matrix of 3D fitted mobile phone camera images using K-means.

**Fig. (16a):**
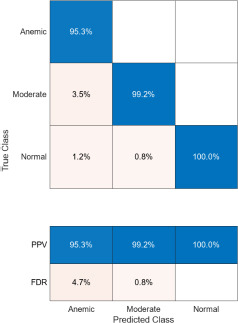
Confusion matrix of DSLR camera images using SAM.

**Fig. (16b):**
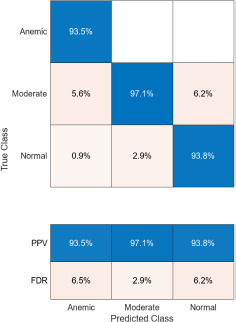
Confusion matrix of mobile phone camera images using SAM.

**Fig. (16c):**
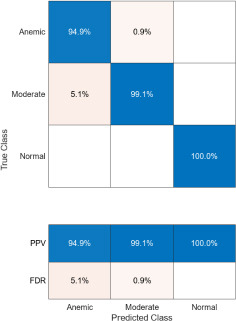
Confusion matrix of 3D fitted mobile phone camera images using SAM.

**Fig. (17a):**
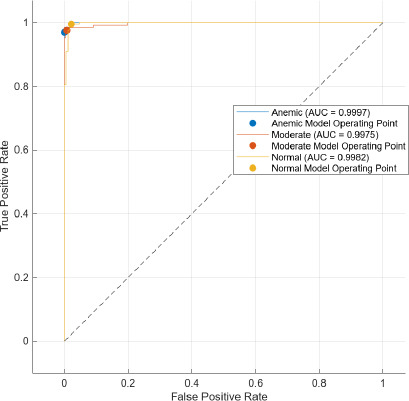
Area under curve model of DSLR camera images using k-means algorithm.

**Fig. (17b):**
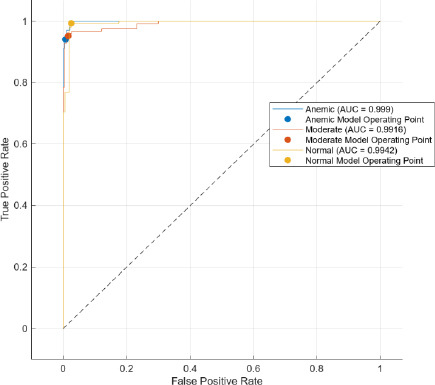
Area under curve model of 3D printed Spacer macro lens fitted Mobile phone camera using K-means.

**Fig. (17c):**
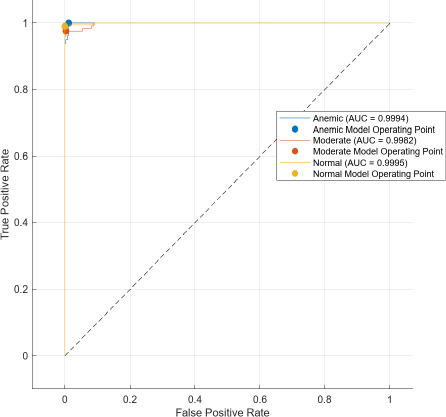
Area Under curve model of DSLR camera images using SAM algorithm.

**Fig. (17d):**
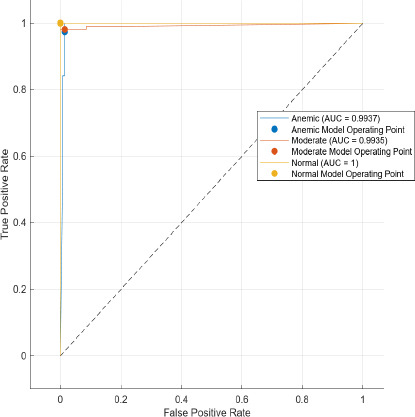
Area Under curve model of 3D printed Spacer macro lens fitted Mobile phone camera images using SAM.

**Fig. (18):**
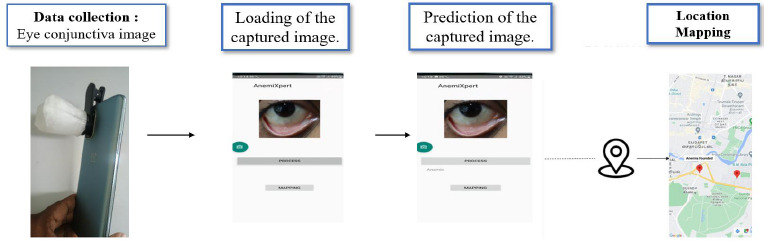
Overall working of android mobile application in screening of anemia.

**Table 1:** Gradation of anemic condition for male and female genders based on hemoglobin level.

**Gender**	**Anemic**	**Moderate Anemic**	**Normal**
**Male**	Less than 10.5g/dL	10.5 g/dL – 13.5 g/dL	More than 13.5g/dL
**Female**	Less than 10.5 g/dL	10.5 g/dL - 12 g/dL	More than 12 g/dL

**Table 2:** YOLO V8 model parameters.

**Model Parameters**	**Values of Parameters**
**Version**	YoloV8m
**Training**	70%
**Validation**	15%
**Testing**	15%
**Epochs**	30
**Learning size**	0.001
**Batch Size**	16

**Table 3:** Segment anything model parameters.

**Model Parameters**	**Values of Parameters**
**Image size**	1024
**Patch size**	16
**Activation function**	Gelu
**Hidden size**	256
**Number Point embedding**	4
**Layer norm epsilon**	le-06
**Mask input channel**	16
**Hidden size**	256
**MLP Dimension**	2048
**Hidden layer**	2
**Attention heads**	8
**IOU Head Depth**	3
**IOU head hidden dimension**	256
**Attention down sample rate**	2

**Table 4:** Model parameters of MLP.

**Model Parameters**	**Values of Parameters**
**Layer size**	25
**Activation function**	ReLu
**Iteration limit**	1000
**No of Fully connected Layer**	1
**Training testing split**	90% 10%
**Validation method**	Resubstitution

**Table 5:** Comparison of classification performance metrics in terms of percentage of DSLR camera, mobile phone camera and 3D-printed spacer macro lens fitted mobile phone camera using (SAM and K-means) algorithm.

**Modality**	**Segmentation Method**	**Accuracy** **(%)**	**Sensitivity** **(%)**	**Specificity** **(%)**	**Precision** **(%)**	**F1-score** **(%)**
**DSLR camera**	K-means	98.5	98	99	99	98.49
-		-	-	-	-	-
**Mobile phone camera**		94.9	95	98	95	95
-		-	-	-	-	-
**3D Printed spacer Macro lens fitted with Mobile phone camera**		98.8	98	98	98	98
-	-	-	-	-	-	-
**DSLR camera**	SAM	98.8	98	98	98	98
-		-	-	-	-	-
**Mobile phone camera**		95	96	98	95	95.49
-		-	-	-	-	-
**3D Printed spacer Macro lens fitted with Mobile phone camera**		98.3	98	99	98	98

**Table 6:** Comparison of research work with existing literature results.

**Approach**	**Number of Classes**	**Segmentation Method**	**Accuracy** **(%)**	**Sensitivity** **(%)**	**Specificity** **(%)**	**Precision** **(%)**	**F1-score** **(%)**	**Reference**
3D Printed spacer* Macro lens assembly attached with smartphone camera	3	K-means	98.8	98	98	98	98	*
3D Printed spacer* Macro lens assembly	3	SAM	98.3	98	99	98	98	*
**Smartphone camera**	2	SLIC	83.3	86.61	-	81.25	84	[5]
**Smartphone camera**	2	HSI color space	88.2	65.21	93.26	-	-	[22]
**Smartphone camera**	2	-	92	-	-	-	-	[31]
**Smartphone camera**	2	-	92.50	90	95	-	-	[32]
**Smartphone camera**	2	-	89.69	90.91	89.06	-	-	[33]
**smartphone camera**	2	-	78.9	-	-	-	-	[34]
**Smartphone camera**	2	K-means	90	-	-	-	-	[35]
*Proposed work	-	-	-	-	-	-	-	-

**Table 7:** Comparison of the cost of the 3D printed spacer macrolens assembly with other commercially available non-invasive anemia detection devices.

**Device**	**Accuracy**	**Cost**	**Approach**
Proposed 3D spacer*	98.3	$1	Non invasive
Masimo pronto	96	$2,500	Non invasive
CBC (Gold standard)	100	$50	invasive

## Data Availability

The data and supportive information are available within the article.

## LIST OF ABBREVIATION

CBC: Complete Blood Count
YOLOv8: You Only Look Once version 8
SAM: Segment Anything Model
MLP: Multi-Layer Perceptron
SDG: Sustainable Development Goals
DSLR: Digital Single-Lens Reflex
ROI: Region of Interest
CNN: Convolutional Neural Network
CSP: Cross-Stage Partial Network
FPN: Feature Pyramid Network
HHR: High Hue Ratio
ReLU: Rectified Linear Unit
ROC: Receiver Operating Characteristic
AUC: Area Under the Curve
PPV: Positive Predictive Value
FDR: False Discovery Rate
FDM: Fused Deposition Modeling
PETG: Polyethylene Terephthalate Glycol
mAP: mean Average Precision
IoU: Intersection over Union
IEC: Institutional Ethics Committee
Hb: Hemoglobin

## STANDARDS OF REPORTING

STROBE guidelines were followed.
